# Contribution of central and peripheral risk factors to prevalence, incidence and progression of knee pain: a community-based cohort study

**DOI:** 10.1016/j.joca.2018.07.013

**Published:** 2018-11

**Authors:** A. Sarmanova, G.S. Fernandes, H. Richardson, A.M. Valdes, D.A. Walsh, W. Zhang, M. Doherty

**Affiliations:** † Division of Rheumatology, Orthopaedics and Dermatology, School of Medicine, University of Nottingham, UK; ‡ Arthritis Research UK Pain Centre, NIHR Nottingham BRC, Nottingham, UK; § Arthritis Research UK Centre for Sports, Exercise and Osteoarthritis, Nottingham, UK

**Keywords:** Knee pain, Risk factors, Cohort, Incidence, Osteoarthritis

## Abstract

**Aim:**

To explore risk factors that may influence knee pain (KP) through central or peripheral mechanisms.

**Methods:**

A questionnaire-based prospective community cohort study with KP defined as pain in or around a knee on most days for at least a month. Baseline prevalence, and one year incidence and progression (KP worsening) were examined. Central (e.g., Pain Catastrophizing Scale (PCS)) and peripheral (e.g., significant injury) risk factors were examined. Adjusted odds ratio (OR) and 95% confidence interval (CI) were calculated using logistic regression. Proportional risk contribution (PRC) was estimated using receiver-operator-characteristic (ROC) analysis.

**Results:**

Of 9506 baseline participants, 4288 (45%) had KP (men 1826; women, 2462). KP incidence was 12% (men 11%, women 13%), and KP progression 19% (men 16%, women 21%) at one year. While both central and peripheral factors contributed to prevalence, central factors contributed more to progression, and peripheral factors more to incidence of KP. For example, although PCS (OR 2.06, 95% CI 1.88–2.25) and injury (5.62, 4.92–6.42) associated with KP prevalence, PCS associated with progression (2.27, 1.83–2.83) but not incidence (1.14, 0.86–1.52), whereas injury more strongly associated with incidence (69.27, 24.15–198.7) than progression (2.52, 1.48–4.30). The PRC of central and peripheral factors were 19% and 23% for prevalence, 14% and 29% for incidence, and 29% and 5% for progression, respectively.

**Conclusions:**

Both central and peripheral risk factors influence KP but relative contributions may differ in terms of development (mainly peripheral) and progression (mainly central). Further study of such relative contributions may inform primary and secondary prevention strategies.

**Key messages**

• Knee pain (KP) is associated with both central and peripheral risk factors
• Peripheral risk factors such as joint injury contribute more to the development of KP
• Central factors such as pain catastrophising and depression appear the main drivers for the progression of KP

## Introduction

Approximately 25% of people aged over 55 have chronic knee pain (KP)^1^. KP is multifactorial and may be caused predominantly by peripheral risk factors such as knee osteoarthritis (OA)^2^, or by alteration in central pain modulatory pathways as in fibromyalgia^3^. KP is a clinical malady related to but not fully explained by knee OA in middle-aged and older adults^4^, ^5^, ^6^. This may explain in part the common discordance between KP and structural knee OA^7^. Risk factors for KP and knee OA may differ, and consideration of both peripheral and central risk factors is important in clinical assessment and to inform the management plan^8^.

KP affects quality of life^9^, causes disability^10^ and associates with increased mortality^11^. Despite treatment attempts an increasing number of people undergo total joint replacement (TJR). However, 15–30% of those undergoing TJR subsequently still experience chronic KP^12^ suggesting that central pain modulation is an important driver of their pain.

The contribution of individual risk factors has been examined previously. However, systematic reviews emphasise that many risk factors have been examined predominantly in cross-sectional studies, and persuasive evidence for causal relationships from cohort studies examining incidence and progression of KP is limited^13^, ^14^, ^15^, ^16^. For example, only a few studies report an association between depression/anxiety and incident KP, and between widespread pain (WSP), previous knee injury and progression of KP^14^, ^16^, ^17^, ^18^, ^19^.

Understanding mechanisms of KP has important implications for targeted management of chronic KP and for predicting response to existing therapies^20^, ^21^, ^22^. The current study aimed to explore risk factors that may influence prevalent KP, incident KP and progression of KP through either peripheral or central mechanisms in a community-based cohort study.

## Methods

This study was approved by Nottingham University Hospitals NHS Trust and the Nottingham Research Ethics Committee 1 (Ref 14/EM/0015).

### Study design and participant selection

Participants were selected from the KP and Related Health in the Community Study (KPIC), an ongoing prospective cohort study aimed to determine the natural history of KP including prevalence, incidence, progression and associated risk factors in community-derived adults^23^. At baseline 40,505 questionnaires were mailed and 9506 men and women aged ≥40 years replied. One year later another questionnaire was posted to the 6716 live participants who indicated willingness to receive further questionnaires. Responders to the Year 1 questionnaire (*n* = 4737) reported a higher frequency of KP and pain severity at baseline compared to non-responders (Appendix 1). Exclusion criteria at baseline were known terminal illness, severe psychiatric illness or dementia, or any other condition or circumstance considered by their General Practice to make them unsuitable to receive the questionnaire.

For the current study we constructed two sub-cohorts from the Year-1 questionnaire responders^1^: an incidence cohort comprising participants with no KP at baseline and therefore at risk of developing new-onset KP (*n* = 2341) during follow-up; and^2^ a progression cohort comprising participants with KP at baseline and therefore at risk for progression (*n* = 2396). An overview of recruitment is presented in Appendix 2.

### Pain measures

***KP*** was defined as pain in or around a knee on most days for at least a month ever^24^. ***Current KP*** was defined as pain on most days of the past month. Prevalence of KP was defined by satisfying the KP criterion at baseline. ***Incident KP*** was defined as no KP at baseline but KP reported at follow-up. ***Worsening of KP*** was defined using the question “Since it has started, do you think the severity of your KP has overall greatly improved/slightly improved/remained the same/worsened” (Patient Global Assessment (PGA)). ***Average pain intensity*** in the past month was assessed on a 0–10 numerical rating scale (NRS).

***Neuropathic-like KP*** was determined using the Pain-DETECT questionnaire modified for use in knee OA (mPDQ score ≥19)^25^. KP was also classified as ***intermittent and/or constant*** using the Measure of Intermittent and Constant Osteoarthritis Pain (ICOAP)^26^. A summative score for each sub-scale was calculated and standardised into 0–100 scale as recommended. Tertiles were calculated for baseline scores of people with KP.

***GP-diagnosed OA*** was self-reported and defined using the question “Have you ever been diagnosed by your doctor as having osteoarthritis of the knee?”

### Risk factors

Age, gender, weight, height and data on other risk factors were included in the questionnaire. Risk factors were divided into three main groups interpreted by the authors as central (related to pain perception and pain physiology), peripheral (related to structural changes in the knee joint) and others (mixed influence).

#### Central risk factors

• ***WSP*** was identified using a diagrammatic manikin and defined as concurrent pain experienced within the past 4 weeks axially, above and below the waist, and on both sides of the body^27^. Current KP was not counted for this.
• ***The Hospital Anxiety and Depression Scale (HADS) and Pain Catastrophizing Scale (PCS) scores*** were each summated as recommended^28^, ^29^. A score on HADS-A or HADS-D ≥8 was used to indicate anxiety or depression^28^. Tertiles of the PCS score were calculated from the whole sample including those with and without KP.

#### Peripheral

• ***A major/significant knee injury*** was defined as a history of knee injury that required seeing a doctor (leg fracture was recorded separately and not included in this definition).
• ***Self-reported frontal plane knee alignment*** was assessed in both knees using a validated line diagram instrument^30^. Participants separately reported their current and early adult life (in their 20's) alignment. Constitutional malalignment was defined as bilateral varus/valgus malalignment (mild or severe) in their 20's.
• ***High risk occupation*** was classified based on published evidence^31^ (Appendix 3). Each listed occupation per individual was analysed and the data dichotomised into high- or low-risk groups.
• ***2D*:*4D*** (index:ring finger length) ratio was self-reported using a validated line-drawing instrument^32^. Type 3 pattern (index shorter than ring finger) is a risk factor for knee OA^32^.

### Other factors

• ***Nodal OA***, which has high heritability and increases risk of knee OA, was determined using a validated line diagram and classified as present in those reporting nodes on at least two rays of each hand^33^.
• ***Comorbidities*:** History or current evidence of comorbidities was recorded for the following: cardiovascular disease (high cholesterol*, heart attack*, angina*, hypertension*), lung disease (chronic obstructive pulmonary disease (COPD), asthma, idiopathic pulmonary fibrosis), endocrine disease (diabetes*, underactive/overactive thyroid, thyroiditis), non-restorative pain disorders (irritable bowel syndrome*, fibromyalgia*, chronic fatigue syndrome*), liver disease (cirrhosis, hepatitis, non-alcoholic fatty liver disease), chronic kidney disease/failure (CKD), central nervous system disorders (stroke*, multiple sclerosis), chronic rheumatic conditions (rheumatoid arthritis, lupus, psoriatic arthritis, ankylosing spondylitis), and gout. In the questionnaire a list of specific conditions (marked *above) and an open “others” question were provided. All conditions were then grouped according to the system as listed above. A comorbidity count was calculated as a total number of affected systems (0–9).
• ***Analgesics*:** Use of prescribed and/or over-the-counter analgesics (e.g., paracetamol; non-steroidal anti-inflammatory drugs (NSAIDs), including COX-2 selective inhibitors; opioids) was self-reported.

## Statistical analysis

### Sample size

Sample size was calculated based on a multivariable logistic regression model^34^, considering 80% power with 5% type I error and the following assumptions^1^: an odds ratio (OR) of 1.4 for a weak but significant association (because a weak association requires larger sample size)^34^; and^2^ a moderate correlation between covariates (*r* = 0.5)^35^. Based on the above assumptions, 452 participants were required for a cross sectional study for the association given a 25% prevalence of KP in the general population^1^, 2548 participants were required for an incidence cohort study according to an annual KP incidence of 3%, and 676 participants were required for a progression cohort study according to an annual rate of worsening of KP of 14%^36^.

### Analysis

The primary focus was to determine multiple risk factors associated with KP. *At baseline* potential risk factors for ***prevalent*** KP were examined using multivariable logistic regression analysis. OR and 95% confidence interval (CI) were estimated with adjustment for age, gender, body mass index (BMI). Adjusting all exposures by the same group of known confounding factors makes adjusted ORs comparable between exposures and would minimize bias related to “Table II Fallacy”^37^. *At follow-up* risk factors for ***incident*** KP or ***worsening*** KP were examined using multivariable logistic regression analysis, all participants having the same one year follow-up period.

**Table I:** Characteristics of the study population (questionnaire survey)

	Baseline			Year-1		
	Total	No KP	KP	Total	Incident KP	Worsening KP
**N**	9506	5218	4288	4737	285	453
**Age (years)**, *mean (SD)*	62.10 (10.56)	62.33 (10.69)	61.82 (10.40)	63.95 (10.13)	62.38 (10.09)	63.03 (10.19)
Age categories						
40–49	1442 (15.17)	793 (15.20)	649 (15.14)	640 (13.51)	39 (13.68)	53 (11.70)
50–59	2297 (24.16)	1210 (23.19)	1087 (25.35)	1112 (23.47)	70 (24.56)	115 (25.39)
60–69	3094 (32.55)	1658 (31.77)	1436 (33.49)	1697 (35.82)	96 (33.68)	157 (34.66)
70–79	2339 (24.61)	1359 (26.04)	980 (22.85)	1167 (24.63)	76 (26.67)	115 (25.39)
≥80	276 (2.90)	162 (3.10)	114 (2.66)	122 (2.57)	4 (1.40)	13 (2.87)
**Women**, *n (%)*	5371 (56.50)	2909 (56.03)	2462 (57.62)	2733 (57.69)	173 (60.70)	290 (64.02)
**BMI (kg/m**^**2**^**)**, *mean (SD)*	27.31 (5.30)	26.36 (4.63)	28.47 (5.80)	27.26 (5.30)	27.36 (4.65)	29.83 (6.28)
**Knee pain, n (%)**	4288 (45.10)	–	4288 (100)	2681 (56.60)	285 (100)	453 (100)
**Current KP, n (%)**	2681 (28.20)	–	2681 (62.52)	1372 (28.96)	175 (61.40)	396 (87.42)
**Current KP severity (NRS 0–10), mean (SD)**	1.97 (2.96)	0.09 (0.60)	4.25 (3.07)	2.06 (2.98)	3.54 (2.61)	6.26 (2.64)
**GP-diagnosed knee OA (%)**	1279 (13.45)	94 (1.80)	1185 (27.64)	722 (15.24)	45 (15.79)	269 (59.38)
**Use of analgesics**						
*Prescribed NSAIDs*, *n (%)*	351 (3.69)	103 (1.97)	248 (5.78)	195 (4.12)	14 (4.91)	44 (9.71)
*Opioids*, *n (%)*	1438 (15.13)	241 (4.62)	646 (15.07)	1077 (22.73)	30 (10.53)	127 (28.04)
*Over-the-counter NSAIDs*, *n (%)*	887 (9.33)	492 (9.43)	946 (22.06)	514 (10.85)	75 (26.32)	211 (46.58)

Abbreviations: SD – standard deviation; NRS – numerical rating scale 0–10; BMI – body mass index; OA – osteoarthritis; NSAIDs – non-steroidal anti-inflammatory drugs; GP -general practitioner.

**Table II:** Risk factors associated with prevalent, incident and worsening knee pain (KP)

	Age, gender, BMI-adjusted ORs (95% CI)		
	Prevalence	Incidence	Worsening
**Central risk factors**			
Widespread pain	**3.02 (2.71; 3.37)**	**2.23 (1.63; 3.06)**	**1.68 (1.35; 2.10)**
HADS anxiety score ≥8	**1.87 (1.71; 2.06)**	1.27 (0.95; 1.70)	**1.68 (1.35; 2.08)**
HADS depression score ≥8	**2.40 (2.14; 2.70)**	**1.99 (1.38; 2.87)**	**1.85 (1.46; 2.34)**
PCS in the highest tertile ≥9)	**2.06 (1.88; 2.25)**	1.14 (0.86; 1.52)	**2.27 (1.83; 2.83)**
mPDQ ≥ 19	**n/a**§	**n/a**§	**2.65 (1.92; 3.65)**
**Peripheral risk factors**			
Significant injury	**5.62 (4.92; 6.42)**	1.08 (0.68; 1.72)	0.89 (0.70; 1.13)
New injury (past 12 months)	**n/a**	**69.27 (24.15; 198.7)**	**2.52 (1.48; 4.30)**
Early varus malalignment	**1.49 (1.18; 1.88)**	1.28 (0.64; 2.57)	1.60 (0.99; 2.58)
Early valgus malalignment	**1.72 (1.25; 2.36)**	1.05 (0.36; 3.12)	1.64 (0.89; 3.03)
Varus malalignment	**5.43 (3.44; 8.58)**	**n/a**	**2.28 (1.30; 4.02)**
Valgus malalignment	**4.18 (2.74; 6.38)**	**n/a**	0.71 (0.37; 1.37)
High risk occupation	**1.43 (1.31; 1.56)**	0.99 (0.76; 1.30)	**1.29 (1.04; 1.60)**
2D4D ratio (type 3)	1.04 (0.95; 1.14)	1.18 (0.90; 1.54)	0.86 (0.69; 1.07)
**Other risk factors**			
Age, mean (SD)∗	1.00 (0.99; 1.00)	1.00 (0.99; 1.01)	1.01 (1.00; 1.02)
Age categories (reference age group 40–49)			
50–59	1.10 (0.95; 1.26)	1.23 (0.80; 1.90)	1.29 (0.88; 1.87)
60–69	1.06 (0.93; 1.21)	1.01 (0.67; 1.53)	1.19 (0.83; 1.71)
70–79	0.93 (0.81; 1.07)	1.20 (0.78; 1.84)	**1.47 (1.01; 2.14)**
≥80	**1.10 (1.01; 1.20)**	0.63 (0.21; 1.84)	1.63 (0.80; 3.35)
Women, *n* (%)†	**1.10 (1.01; 1.20)**	1.15 (0.89; 1.50)	**1.34 (1.07; 1.66)**
BMI (kg/m^2^), mean (SD)‡	**1.08 (1.07; 1.09)**	**1.06 (1.03; 1.09)**	**1.05 (1.04; 1.07)**
Nodal OA	**1.89 (1.64; 2.17)**	1.49 (0.99; 2.23)	**1.62 (1.23; 2.14)**
ICOAP intermittent in the highest tertile (≥7)	**n/a**§	**n/a**§	**3.61 (2.89; 4.50)**
ICOAP constant in the highest tertile (≥7)	**n/a**§	**n/a**§	**3.70 (2.96; 4.63)**
N of comorbidities	**1.21 (1.15; 1.28)**	**1.31 (1.12; 1.53)**	1.13 (1.00; 1.27)
Any comorbidities	**1.29 (1.18; 1.42)**	**1.48 (1.12; 1.95)**	**1.29 (1.02; 1.64)**
Any comorbidities ≥ 2	**1.37 (1.22; 1.53)**	1.26 (0.90; 1.78)	1.26 (0.98; 1.61)
Any comorbidities ≥ 3	**1.68 (1.34; 2.11)**	**2.55 (1.41; 4.60)**	0.98 (0.62; 1.54)
Any comorbidities ≥ 4	1.51 (0.80; 2.84)	**8.85 (1.95; 40.16)**	0.70 (0.15; 3.18)

In bold - If a 95% confidence interval does not include the null value (i.e. there is a statistically meaningful or statistically significant difference between the groups).

**Abbreviations:** BMI - body mass index; KP – knee pain; OR – odds ratio; CI –confidence interval; HADS - Hospital Anxiety and Depression Scale; PCS - Pain Catastrophizing Scale; SD - standard deviation; PDQ – Pain DETECT questionnaire; ICOAP - Measure of Intermittent and Constant Osteoarthritis Pain.

∗ Adjusted for gender and BMI only.

† Adjusted for age and BMI only.

‡ Adjusted for age and gender only.

§ mPDQ and ICOAP scores (intermittent and constant) were knee-specific, so if there was no KP at baseline, the score was 0 and therefore could not be a risk factor for prevalent or incident KP.

Sensitivity analysis was undertaken using an alternative definition of KP worsening, defined by a significant increase from baseline in KP severity on NRS. A significant change from the baseline was calculated using the least significant criterion (LSC)^38^.

We used receiver-operator-characteristic (ROC) curves and areas under the curve (AUC) to examine proportional risk contribution (PRC) of central and peripheral risk factors. ROC curves were based on the multivariable logistic regression model with prevalent/incident/worsening KP as an outcome. Firstly, we built the full risk model for each outcome with a ROC curve (AUC_f_). Secondly, we removed the exposure(s) of interest to examine the contribution of the exposure (s) removed through the reduction of the ROC curve, i.e., the partial AUC (AUC_p_). Thirdly we calculated the PRC using the following formula: $PRC=\frac{AUC_{f}-AUC_{p}}{AUC_{f}-0.5}$, where 0.5 is the AUC under the diagonal line of the ROC curve indicating no discrimination at all by all included risk factors. The full model included central (WSP, HADs, PCS), peripheral (history of significant injury, early life mal-alignment, current mal-alignment, high risk occupation) and other risk factors (age, gender, BMI, number of comorbidities, nodal hand osteoarthritis). In addition, the full model for incident and worsening KP included new knee injury (past 12 months) as a peripheral risk factor.

The described models for PRC are different from the models used to estimate associations (age, gender and BMI adjusted) because of the different purposes of the two models. While the latter model aimed to make a comparison between different risk factors, the models used to estimate PRC aimed to examine the contribution of central and peripheral risk factors in the context of all possible risk factors.

All analyses were undertaken using SAS 9.4.

## Results

### Demographics of participants

Characteristics of the study population are presented in Table I. The baseline mean age was 62.1 (range 40–86) years, 57% were women, and the mean BMI was 27.31 (SD 5.30, range 13–74). Prevalence of KP was 45% (men, 44%; women, 46%), prevalence of current KP was 28% (men 27%, women 29%), and 14% had GP-diagnosed OA (men 12%, women 15%). People with KP used more NSAIDs and opioids than those without (6% vs 2% for prescribed NSAIDs, 22% vs 9% for over-the-counter NSAIDs, 15% vs 5% for opioids). More than half the study population reported one or more comorbidities (61% of people with KP, 53% of those without). The most prevalent conditions in KP participants were cardiovascular diseases (54%), non-restorative pain disorders (16%), and endocrine diseases (15%). Detailed comorbidity results are presented in Appendix 4.

Incidence of KP during the 1 year follow-up was 12% (285/2341). This was similar in men (11%) and women (13%). During the one year follow-up, 19% (453/2396) reported worsening of KP (men 16%, women 21%).

### Risk factors for prevalent KP

Both central and peripheral risk factors associated with KP (Table II, Appendix 5). Among central factors, WSP was the strongest (OR 3.02, 95% CI 2.71, 3.37), followed by depression (OR 2.40, 95% CI 2.14, 2.70 for HAD-D≥8), pain catastrophizing (OR 2.06, 95% CI 1.88, 2.25 for PCS≥9) and anxiety (OR 1.87, 95% CI 1.71, 2.06 for HAD-A≥8). Among peripheral factors, previous knee injury was the strongest (OR 5.62, 95% CI 4.92, 6.42), followed by constitutional mal-alignment (OR 1.49, 95%CI 1.18, 1.88 for varus, and OR 1.72, 95%CI 1.25, 2.36 for valgus) and high risk occupation (OR 1.43, 95% CI 1.31, 1.56). Among other risk factors nodal hand OA (OR 1.89, 95% CI 1.64, 2.17), comorbidity (OR 1.29, 95% CI 1.18, 1.42 for any comorbidity), gender and BMI associated with KP.

**Fig. 1:**
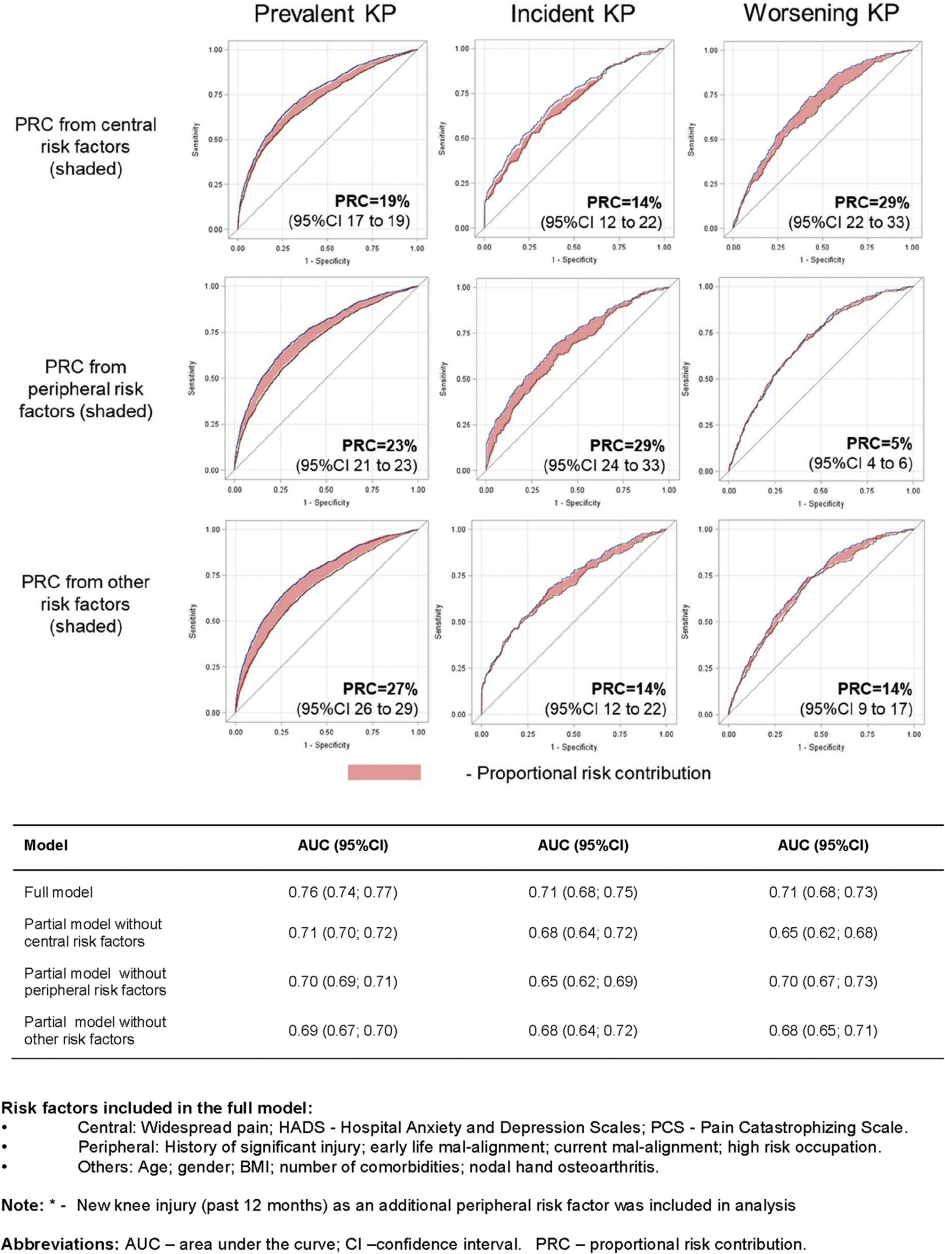
Proportional risk contribution (PRC) to prevalence, incidence and progression of knee pain (KP).

The AUC for the full model including central, peripheral and other factors was 0.76 (0.74, 0.77). The PRC of central and peripheral factors to the full model was 19% and 23%, respectively, and contribution of other factors was 27% (Fig. 1). Detailed ROC analyses and relative contributions of central and peripheral risk factors are shown in Appendix 6.

### Risk factors for incident KP

The strongest central factors associated with incident KP were WSP (OR 2.23, 95% CI 1.63, 3.06) and depression (OR 1.99, 95% CI 1.38, 2.87 for HAD-D≥8). Peripheral risk factors (knee injury reported at baseline and constitutional mal-alignment) had no significant effect on risk of incident KP. However, new knee injury during follow-up was a strong risk factor for KP onset (OR 69.27, 95%CI 24.15, 198.7). BMI and presence of any comorbidity were also risk factors for incident KP. The relationship of each baseline risk factor to development of incident KP is presented in Table II and Appendix 7.

The PRC of central and peripheral risk factors to incident KP was 14% and 29% respectively (Fig. 1).

### Risk factors for KP worsening

In people with KP both central and peripheral risk factors contributed to KP worsening during 1 year. The following central factors associated with worsening: pain catastrophising (OR 2.27, 95%CI 1.83, 2.83 PCS≥9), depression (OR 1.85, 95% CI 1.46, 2.34), anxiety (OR 1.68, 95% CI 1.35, 2.08) and WSP (OR 1.68, 95% CI 1.35, 2.10). In addition, those with neuropathic-like pain and higher ICOAP scores (both intermittent and constant) were more likely to report worsening (Table II, Appendix 8). With respect to peripheral factors, significant injury before baseline did not associate with worsening KP, whereas injury during follow-up) did (OR 2.52, 95% CI 1.48, 4.30). Current varus mal-alignment (OR 2.28, 95% CI 1.30, 4.02) and high risk occupation also associated. Other risk factors such as nodal hand OA (OR 1.62, 95% CI 1.23, 2.14), presence of any comorbidity (OR 1.29, 95% CI 1.02, 1.64) and BMI associated with KP worsening.

The sensitivity analysis using an alternative definition of KP worsening showed that only nodal OA and high risk occupation predicted increase in KP severity from baseline (Appendix 9).

Overall, the contribution from peripheral factors to KP worsening was much smaller (PRC = 5%) than that from central factors (PRC = 29%) (Fig. 1).

## Discussion

This is the first community-based cohort study to investigate the contribution of central and peripheral risk factors associated with KP prevalence, incidence and progression. Central factors were those related to pain perception, psychological and behavioural response that could affect normal pain physiology^39^, while peripheral factors were those related to structural changes in the knee. Some risk factors such as comorbidities and nodal hand OA could influence KP through both central and peripheral mechanisms so were classified as “other” risk factors. The main findings are^1^: both central and peripheral risk factors associated with *prevalence of KP*^2^; peripheral risk factors such as knee injury contributed more to *incidence of KP*; and^3^ central risk factors such as PCS contributed more to KP *progression*.

We examined central (e.g., WSP, anxiety, depression, catastrophizing), peripheral (e.g., injury and mal-alignment) and other risk factors and confirm that many of these associate with KP (Table II). Some, such as WSP and comorbidities appear to act particularly as risk factors for developing KP, whereas others such as neuropathic-like pain, pain catastrophizing and current varus mal-alignment may in part result from KP and particularly associate with pain progression. Furthermore, localised KP or OA might have reciprocal effects on incident depression or WSP which later contribute to worsening of symptoms^40^, ^41^, ^42^. For each outcome (prevalent, incident and worsening of KP) we performed a two-stage analysis. Firstly, we examined the association between independent risk factors and KP using OR as a measure of association. Secondly, we explored the relative contribution of risk factors in composite divided into three main groups (central, peripheral and others) using PRC.

The positive association between depression and KP worsening accords with two large longitudinal studies of people with KP and knee OA^43^, ^44^. The contribution of anxiety to KP worsening has been reported previously by Mallen *et al*. (2007)^45^. In a study by Jinks *et al*. (2008) WSP (two or more pain sites) was associated with onset but not progression of KP^17^. However, our study showed a strong association with KP for WSP and depression in the cross-sectional analysis, and also convincing evidence of a causal relationship from the two sub-cohort studies of incidence and progression of KP in one year. A consistent association between prevalence, incidence or worsening of KP and central risk factors highlights the important contribution of centrally-mediated mechanisms to the natural history of KP, supporting the concept that KP shares risk factors in common with other chronic pain disorders such as fibromyalgia and low back pain^46^, ^47^.

Major knee trauma is a recognised important risk factor for knee OA onset^14^. In our study, previous knee injury was a strong risk factor for KP in the cross-sectional analysis, and new injury associated with both incidence and progression of KP (Table II). However, the association between some peripheral factors and KP in our study did not always replicate findings from some previous cohort (mainly knee OA) studies. For example, although constitutional (early life) varus/valgus mal-alignment was a risk factor for incident KP in a study by Ingham *et al*. (2011)^48^, we found no association between constitutional malalignment and incidence or progression of KP. The literature about effect of constitutional mal-alignment on KP is limited^15^. However, our findings that current knee mal-alignment, which is more likely a consequence of knee OA, is a stronger predictor for progression of KP accords with other literature^15^, ^49^.

We additionally examined the relative contribution of central and peripheral risk factors in a full risk model using a proportion derived from ROC curve analysis (PRC). For incident KP the contribution of peripheral factors was larger than that of central factors (29% vs 14%), whereas for worsening KP the contribution of central factors was larger than that of peripheral factors (29% vs 5%). This suggests that the relative contribution of central and peripheral risk factors to KP may differ at different stages of the condition (development or progression). Incident KP is not often centrally driven, however the influence of central risk factors (existing before or developing later due to chronic pain) increases as the condition progresses^50^, ^51^.

There are a number of caveats to this study. Firstly, the overall prevalence of KP in this population (45%) was slightly higher than most previous studies (e.g., 28% in a previous Nottingham study^24^). This may reflect response bias in that people with KP may more likely respond to a KP questionnaire. Indeed comparison between responders and non-responders to the Year-1 questionnaire showed that people with KP and those with higher pain severity were more likely to respond (Appendix 2). Secondly, previous studies showed that the question we used to define KP is the most sensitive (58.7%) but least specific (59.1%) predictor of grade ≥1 osteophytes^24^ and applying a different KP definition might give different results. Thirdly, pain and risk factors were measured at just two time points and repeated measures over a longer period might reveal different trajectories of KP progression and better determination of associations. Fourthly, both definitions for worsening were self-reported and may have limitations. PGA reflects the perception of worsening (i.e., retrospective perspective by the participant at the time of Y1 assessment) for the full course of the disease but the risk factors captured at baseline may have changed since the start of the disease (Table II). In contrast, NRS was measured at only two time points during a short time period - one year, which cannot reflect the actual pain trajectory. Fifthly, we interpret our data as showing risk from central and peripheral factors, but cannot exclude possible confounding by other factors. Furthermore, the number of peripheral factors studied was limited. The use of imaging and clinical assessments would have allowed more detailed assessment of abnormal joint features and changes in knee alignment over time. However, this would not affect the examination of the relative contribution of the questionnaire based surrogate measures of peripheral risk factors in different stages of KP. Finally, this is the first time that we have used this measure for PRC of central or peripheral risk factors. A caveat of this measure is that it increases with number of risk factors collected. However, when we compare the same group of risk factors across different stages of a disease, e.g., early (incidence) and later (progression) stages of KP, relative PRCs can be determined. As with the original measure of AUC, the derived measure of PRC is also open to bias due to model overfitting especially when the sample size is small. However, this study involved a large sample of 9506 community-derived participants so overfitting is unlikely to be an important issue^52^.

In conclusion, this study confirms that a number of peripheral and central risk factors associate with prevalence of KP. While peripheral risk factors such as joint injury appear the main drivers for development of KP, central factors such as PCS appear the main drivers for KP progression. Further study is needed to examine the contribution of other peripheral risk factors such as radiographic change, synovial changes and muscle strength, as well as more objective central risk factor measures such as quantitative sensory testing and neuroimaging.

## Authors' contributions

AS, WZ and MD made substantial contributions to the conception and design of the study. All authors contributed to the acquisition of questionnaire data. AS, MD and WZ conducted the data analysis and interpretation. AS wrote the first draft. WZ has full access to the data and takes responsibility for the content and guarantees the integrity and accuracy of the work undertaken. All authors have read, provided critical feedback on intellectual content and approved the final manuscript.

## Declaration of Helsinki

This study complies with the Declaration of Helsinki and was approved by Nottingham University Hospitals NHS Trust and the Nottingham Research Ethics Committee 1 (Ref 14/EM/0015).

## Conflict of interest disclosures

All authors have completed and submitted the ICMJE Form for Disclosure of Potential Conflicts of Interest.

## Funding/support

This study was supported financially by the Arthritis Research UK Pain Centre (Centre Initiative grant number 20777) and Arthritis Research UK Centre for Sport, Exercise and Osteoarthritis (Grant reference 20194). The authors would also like to thank the Bolashak scholarship programme, offered by the Ministry of Education and Science of the Republic of Kazakhstan, for financially supporting the PhD research programme (AS) and the University of Nottingham as sponsor and host institution of this project.

## Role of the funder**/**sponsor

The sponsor did not participate in the design and conduct of the study; collection, management, analysis, and interpretation of the data; or preparation, review, or approval of the manuscript and the decision to submit the manuscript for publication.

## Disclaimer

The opinions, results and conclusions reported in this article are those of the authors and are independent from the funding sources.

## Figures and Tables

**Table:** 

	Non-Responders (*N* = 4769)	Responders (*N* = 4737)	*P*-value∗
Age (years), mean (SD)	61.73 (10.96)	62.47 (10.14)	0.0011
Women, *n* (%)	2631 (55.17)	2740 (57.84)	0.0291
BMI, mean (SD)	27.37 (5.30)	27.26 (5.30)	0.3164
Pain severity (NRS 0–10)†, mean (SD)	1.78 (2.90)	2.15 (3.00)	<0.0001
Knee pain, *n* (%)	1892 (39.67)	2396 (50.58)	<0.0001
GP-diagnosed knee OA, *n* (%)	557 (11.68)	722 (15.24)	<0.0001
Widespread pain	960 (20.13)	1110 (23.43)	0.0001
HADS anxiety score ≥8	1623 (34.04)	1460 (30.81)	0.0008
HADS depression score ≥8	940 (19.71)	767 (16.19)	<0.0001
PCS in the highest tertile (≥9)	1647 (34.56)	1609 (33.97)	0.5441
mPDQ ≥ 19	184 (3.86)	202 (4.26)	0.3180
Significant injury	662 (14.48)	889 (19.18)	<0.0001
Early varus malalignment	141 (3.40)	174 (3.98)	0.1594
Early valgus malalignment	95 (2.31)	87 (2.02)	0.3596
Varus malalignment	72 (1.68)	72 (1.60)	0.7605
Valgus malalignment	77 (1.80)	87 (1.93)	0.6434
High risk occupation	2053 (43.06)	1926 (40.65)	0.0173
2D4D ratio (type 3)	2124 (48.59)	2360 (51.93)	0.0017
Nodal OA	471 (10.40)	589 (12.92)	0.0002
ICOAP intermittent in the highest tertile (≥7)	664 (13.93)	835 (17.62)	<0.0001
ICOAP constant in the highest tertile (≥7)	699 (14.66)	792 (16.72)	0.0059

**Abbreviations:** SD - standard deviation; BMI - body mass index; NRS – numerical rating scale (range 0–10).

∗ *t*-test for continuous and chi-square test for categorical.

† Average pain severity in the past month.

**Figure:**
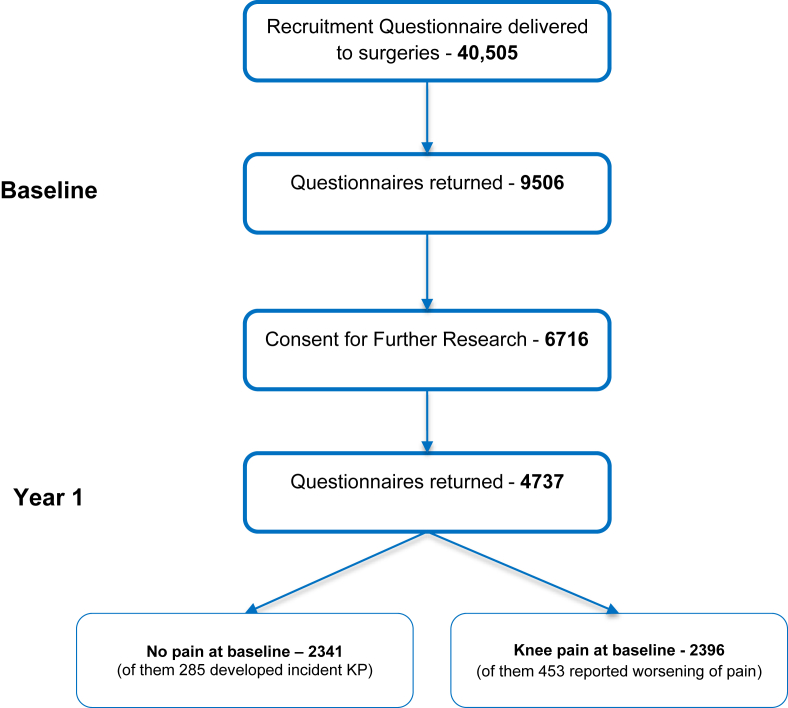


**Table:** 

Agreed high risk category	Filter searches	References
Auto-industry/mechanic	Car Fitter, Car Seat Assembly, Paint Sprayer/Car Repair (seat assembler, reconditioning, painting), car mechanic, car repairer, Panel beater	^1^
Tradesmen: construction	Brick, Build, Property, Construction, Scaffold, Damp, Ground worker, Quarry worker/operative, roof, Joiner, Carpenter, Wood, Fitter, Sign maker, Furniture design and manufacture, Upholsterer, Cabinet Maker, Shop Fitter, Window, Glazing, Glazier, Double Glazier	^1,2,3^
Tradesmen: other	Electric, Jointer Electrical, Lineman, Panel wirer/wireman, Carpet, Floor, Slab, Paint, Plaster, Cabinet Sprayer, Plumber, Pipe Fitter	^1,2,3^
Emergency services	Police, Firefighter, Army, Soldier, National Service, RAF, Marines, Navy, Naval, Paratrooper, Royal (air force etc.), Ambulance, Paramedic, Military, WRNS (Women's Royal Navy Service)	
Factory/warehouse worker	Factory, Workshop, Worker, Assembly, Production, Warehouse, Picker, Packer	^4^
Land management/cultivation	Agriculture, Agricultural, Farm, Farmer, “Picker (fruits)”, Herdsman, Dairy man, Gardener, Green, Landscape, Lawn, Horticulture, Groundsman, Greenkeeper, Horticulturalist, Timber yard worker, forest worker, forester, forestry	^1,2^
Manual workers engineering	Engineer, Millwright, Lagger (Insulators)/Lagging	^3^
Health Services	Nurse, Nursing, carer, care worker, care assistant, support worker, Home help, RGN (registered general nurse), RNMH (registered nurse mental health), Ward Orderly, SRN (State Registered Nurse), RMN (Registered Mental Nurse), RNLD	^2,5^
Coal miner	Coal, Mine, Colliery, Down pit, Mining, Collier	^3^
Metal worker	Steel, Weld, Metal, Blacksmith, Foundry, Moulder, Plater, Fabricator	^1^
PCM (Porters, cleaners, maintenance)	Porter, Clean Window, Cleaner, Valeter, Wash (Car), Janitor, Domestic Assistant, Chambermaid, Hospital domestic, domestic, caretaker, building maintenance, maintenance, handyman, building assistant	^1,3^
PE teacher	PE, Physical	^3^
Postman	Post, mail	Agreed in Consensus Meeting due to prolongued walking, bending and lifting.
Labourer	Labourer, Pipe yard Worker, Concrete, Installer, Mason, Fibre glass, Asphalt(er), Digging trenches, Fencer, Manual	^4^^,6^
Road worker	Roadworks, Road, Highway, Platelayer, Tarmac layer, Tarmacer	^4^
Shipyard worker	Shipwright	^1,3^
Sports-other	Any, Climbing instructor, Parachute instructor	

^1^ Coggon D, Croft P, Kellingray S, et al. Occupational physical activities and osteoarthritis of the knee. Arthritis and rheumatism 2000;43(7):1443-9. doi: 10.1002/1529-0131(200007)43:7<1443::aid-anr5>3.0.co;2-1 [published Online First: 2000/07/21]

^2^ Andersen S, Thygesen LC, Davidsen M, et al. Cumulative years in occupation and the risk of hip or knee osteoarthritis in men and women: a register-based follow-up study. Occup Environ Med 2012;69(5):325-30. doi: 10.1136/oemed-2011-100033 [published Online First: 2012/01/14]

^3^ Pensions IIACaDfWa. Disorders of the knee: report by the Industrial Injuries Advisory Council, 1995.

^4^ Palmer KT. Occupational activities and osteoarthritis of the knee. British medical bulletin 2012;102:147-70. doi: 10.1093/bmb/lds012

^5^ Companies DS. Hardest Working Knees: Occupational Activities That Can Cause or Aggravate Knee Osteoarthritis. Information Sheet 2015 [Available from: https://www.depuysynthes.com/binary/org/DPY_SYN/knee/campaigns/HWK_Backgrounder_12_02_15FINAL.pdf.]

^6^ Lindberg H, Montgomery F. Heavy labor and the occurrence of gonarthrosis. Clinical orthopaedics and related research 1987(214):235-36.

**Table:** 

	Total	No KP (Q11)	Knee pain (Q11)	*P*-value
**N**	9506	5218	4288	
**Conditions**				
*High cholesterol*, *n (%)*	2634 (27.71)	1356 (26.00)	1278 (29.80)	<0.0001
*Heart attack/angina*, *n (%)*	604 (6.35)	287 (5.50)	317 (7.39)	0.0002
*Hypertension*, *n (%)*	2845 (29.93)	1400 (26.85)	1445 (33.70)	<0.0001
*Diabetes*, *n (%)*	899 (9.46)	423 (8.11)	476 (11.10)	<0.0001
*Underactive/overactive thyroid*, *thyroiditis*, *n (%)*	369 (3.88)	185 (3.54)	184 (4.29)	0.0608
*Stroke*, *n (%)*	271 (2.85)	142 (2.72)	129 (3.01)	0.4045
*Multiple sclerosis*, *n (%)*	18 (0.19)	10 (0.19)	8 (0.19)	0.9551
*Irritable bowel syndrome*, *n (%)*	963 (10.13)	427 (8.19)	536 (12.50)	<0.0001
*Fibromyalgia*, *n (%)*	246 (2.59)	68 (1.30)	178 (4.15)	<0.0001
*Chronic fatigue syndrome*, *n (%)*	133 (1.40)	55 (1.05)	78 (1.82)	0.0016
*Chronic obstructive pulmonary disease (COPD)*, *n (%)*	103 (1.08)	54 (1.03)	49 (1.14)	0.6126
*Asthma*, *n (%)*	412 (4.33)	212 (4.06)	200 (4.66)	0.1514
*Idiopathic pulmonary fibrosis*, *n (%)*	3 (0.03)	3 (0.06)	0 (0.00)	0.1164
*Liver cirrhosis*, *n (%)*	6 (0.06)	4 (0.08)	2 (0.05)	0.5622
*Hepatitis*, *n (%)*	5 (0.05)	2 (0.04)	3 (0.07)	0.5031
*Non-alcoholic fatty liver disease*, *n (%)*	6 (0.06)	4 (0.08)	2 (0.05)	0.5622
*Rheumatoid arthritis*, *n (%)*	92 (0.97)	25 (0.48)	67 (1.56)	<0.0001
*Ankylosing spondylitis*, *n (%)*	19 (0.20)	3 (0.06)	16 (0.37)	0.0006
*Lupus*, *n (%)*	12 (0.13)	4 (0.08)	8 (0.19)	0.1331
*Psoriatic arthritis*, *n (%)*	17 (0.18)	4 (0.08)	13 (0.30)	0.0093
**Systems affected**				
Cardiovascular disease, *n* (%)	4124 (43.38)	2105 (40.34)	2019 (47.08)	<0.0001
Endocrine disease, *n* (%)	1229 (12.93)	595 (11.40)	634 (14.79)	<0.0001
Non-restorative pain disorders, *n* (%)	1164 (12.24)	497 (9.52)	667 (15.56)	<0.0001
Lung disease, *n* (%)	497 (5.23)	263 (5.04)	234 (5.46)	0.3624
Liver disease, *n* (%)	17 (0.18)	10 (0.19)	7 (0.16)	0.7447
Chronic kidney disease/failure (CKD), *n* (%)	23 (0.24)	8 (0.15)	15 (0.35)	0.0523
Central nervous system disorders, *n* (%)	289 (3.04)	152 (2.91)	137 (3.19)	0.4246
Gout, *n* (%)	94 (0.99)	54 (1.03)	40 (0.93)	0.6175
Chronic rheumatic conditions, *n* (%)	138 (1.45)	35 (0.67)	103 (2.40)	<0.0001
**Comorbidity count**				
N of comorbidities, mean (SD), *n* (%)	0.80 (0.85)	0.71 (0.81)	0.90 (0.90)	<0.0001
Any comorbidities, *n* (%)	5355 (56.33)	2740 (52.51)	2615 (60.98)	<0.0001
Any comorbidities ≥ 2, *n* (%)	1804 (18.98)	822 (15.75)	982 (22.90)	<0.0001
Any comorbidities ≥ 3, *n* (%)	367 (3.86)	137 (2.63)	230 (5.36)	<0.0001
Any comorbidities ≥ 4, *n* (%)	45 (0.47)	19 (0.36)	26 (0.61)	0.0868

**Table:** 

	Descriptive			ORs (95% CI)	
	Total	No KP	KP	Crude	Age, gender, BMI-adjusted
**N**	9506	5218	4288		
**Central risk factors**					
Widespread pain, *n* (%)	2070 (21.77)	683 (13.09)	1387 (32.35)	3.17 (2.86; 3.52)	**3.02 (2.71; 3.37)**
HADS anxiety score ≥8, *n* (%)	3083 (32.43)	1349 (25.85)	1734 (40.44)	1.95 (1.79; 2.12)	**1.87 (1.71; 2.06)**
HADS depression score ≥8, *n* (%)	1707 (17.96)	593 (11.36)	1114 (25.98)	2.74 (2.45; 3.05)	**2.40 (2.14; 2.70)**
PCS in the highest tertile (≥9), *n* (%)	3256 (34.25)	1354 (25.96)	1902 (44.37)	2.27 (2.09; 2.48)	**2.06 (1.88; 2.25)**
**Peripheral risk factors**					
Significant injury, *n* (%)	1551 (16.31)	352 (6.97)	1199 (28.83)	5.40 (4.76; 6.14)	**5.62 (4.92; 6.42)**
Early varus malalignment, *n* (%)	315 (3.31)	147 (3.18)	168 (4.31)	1.37 (1.09; 1.72)	**1.49 (1.18; 1.88)**
Early valgus malalignment, *n* (%)	182 (1.91)	71 (1.56)	111 (2.88)	1.87 (1.38; 2.53)	**1.72 (1.25; 2.36)**
Varus malalignment, *n* (%)	144 (1.51)	23 (0.49)	121 (2.99)	6.31 (4.03; 9.88)	**5.43 (3.44; 8.58)**
Valgus malalignment, *n* (%)	164 (1.73)	35 (0.74)	129 (3.19)	4.42 (3.03; 6.44)	**4.18 (2.74; 6.38)**
High risk occupation, *n* (%)	3979 (41.85)	1973 (37.80)	2006 (46.78)	1.45 (1.33; 1.57)	**1.43 (1.31; 1.56)**
2D4D ratio (type 3), *n* (%)	4484 (47.17)	2439 (49.86)	2045 (50.81)	1.04 (0.96; 1.13)	1.04 (0.95; 1.14)
**Other risk factors**					
Age, mean (SD)∗	62.10 (10.56)	62.33 (10.69)	61.82 (10.40)	1.00 (0.99; 1.00)	1.00 (0.99; 1.00)
Women, *n* (%)†	5371 (56.50)	2909 (56.03)	2462 (57.62)	1.07 (0.98; 1.16)	**1.10 (1.01; 1.20)**
BMI (kg/m^2^), mean (SD)‡	27.31 (5.30)	26.36 (4.63)	28.47 (5.80)	1.08 (1.07; 1.09)	**1.08 (1.07; 1.09)**
Nodal OA, *n* (%)	1060 (11.15)	445 (8.86)	615 (15.11)	1.83 (1.61; 2.08)	**1.89 (1.64; 2.17)**
N of comorbidities, mean (SD), *n* (%)	0.80 (0.85)	0.71 (0.81)	0.90 (0.90)	1.29 (1.23; 1.36)	**1.21 (1.15; 1.28)**
Any comorbidities, *n* (%)	5355 (56.33)	2740 (52.51)	2615 (60.98)	1.41 (1.30; 1.53)	**1.29 (1.18; 1.42)**
Any comorbidities ≥ 2, *n* (%)	1804 (18.98)	822 (15.75)	982 (22.90)	1.59 (1.43; 1.76)	**1.37 (1.22; 1.53)**
Any comorbidities ≥ 3, *n* (%)	367 (3.86)	137 (2.63)	230 (5.36)	2.10 (1.69; 2.61)	**1.68 (1.34; 2.11)**
Any comorbidities ≥ 4, *n* (%)	45 (0.47)	19 (0.36)	26 (0.61)	1.67 (0.92; 3.03)	1.51 (0.80; 2.84)

In bold - If a 95% confidence interval does not include the null value (i.e. there is a statistically meaningful or statistically significant difference between the groups).

**Abbreviations:** BMI - body mass index; KP – knee pain; OR – odds ratio; CI –confidence interval; HADS - Hospital Anxiety and Depression Scale; PCS - Pain Catastrophizing Scale; SD – standard deviation.

∗ Adjusted for gender and BMI only.

† Adjusted for age and BMI only.

‡ Adjusted for age and gender only.

**Table:** 

Outcome	Model	AUC (95%CI)	PRC
Prevalent KP	Full	0.76 (0.74; 0.77)	100%
	*without central*	0.71 (0.70; 0.72)	19%
	*without peripheral*	0.70 (0.69; 0.71)	23%
	*without others*	0.69 (0.67; 0.70)	27%
Incident KP	Full∗	0.71 (0.68; 0.75)	100%
	*without central*	0.68 (0.64; 0.72)	14%
	*without peripheral*	0.65 (0.62; 0.69)	29%
	*without others*	0.68 (0.64; 0.72)	14%
Worsening KP	Full∗	0.71 (0.68; 0.73)	100%
	*without central*	0.65 (0.62; 0.68)	29%
	*without peripheral*	0.70 (0.67; 0.73)	5%
	*without others*	0.68 (0.65; 0.71)	14%

**Risk factors included in the full model:**

• Central: Widespread pain; HADS - Hospital Anxiety and Depression Scales; PCS - Pain Catastrophizing Scale.

• Peripheral: History of significant injury; early life mal-alignment; current mal-alignment; high risk occupation.

• Others: Age; gender; BMI; number of comorbidities; nodal hand osteoarthritis.

**Abbreviations:** AUC – area under the curve; CI –confidence interval. PRC – proportional risk contribution.

∗ New knee injury (past 12 months) as an additional peripheral risk factor was included in analysis.

**Table:** 

	Descriptive			OR (95% CI)	
	Total	No KP	Incident KP	Crude	Age, gender, BMI-adjusted
**N**	2341	2056	285		
**Central risk factors**					
Widespread pain at baseline, *n* (%)	316 (13.50)	247 (12.01)	69 (24.21)	2.34 (1.73; 3.16)	**2.23 (1.63; 3.06)**
HADS anxiety score ≥8 at baseline, *n* (%)	559 (23.88)	477 (23.20)	82 (28.77)	1.34 (1.02; 1.76)	1.27 (0.95; 1.70)
HADS depression score ≥8 at baseline, *n* (%)	216 (9.23)	169 (8.22)	47 (16.49)	2.21 (1.55; 3.13)	**1.99 (1.38; 2.87)**
PCS in the highest tertile (≥9) at baseline, *n* (%)	611 (26.1)	524 (25.50)	87 (30.63)	1.29 (0.98; 1.69)	1.14 (0.86; 1.52)
**Peripheral risk factors**					
Significant injury at baseline, *n* (%)	183 (7.82)	158 (7.85)	25 (8.90)	1.15 (0.74; 1.78)	1.08 (0.68; 1.72)
New injury (past 12 months), *n* (%)	38 (1.62)	4 (0.19)	34 (11.93)	69.49 (24.46; 197.4)	**69.27 (24.15; 198.7)**
Early varus malalignment, *n* (%)	72 (3.07)	62 (3.25)	10 (3.86)	1.20 (0.61; 2.36)	1.28 (0.64; 2.57)
Early valgus malalignment, *n* (%)	28 (1.20)	23 (1.23)	5 (1.96)	1.60 (0.60; 4.26)	1.05 (0.36; 3.12)
High risk occupation, *n* (%)	833 (35.57)	732 (35.59)	101 (35.44)	0.99 (0.77; 1.29)	0.99 (0.76; 1.30)
2D4D ratio (type 3), *n* (%)	1171 (50.02)	1023 (51.10)	148 (54.81)	1.16 (0.90; 1.50)	1.18 (0.90; 1.54)
**Other risk factors**					
Age (years), mean (SD)∗	62.54 (10.25)	62.56 (10.28)	62.38 (10.09)	1.00 (0.99; 1.01)	1.00 (0.99; 1.01)
Women, *n* (%)†	1343 (57.37)	1170 (56.93)	173 (60.70)	1.17 (0.91; 1.51)	1.15 (0.89; 1.50)
BMI (kg/m^2^), mean (SD)‡	26.20 (4.50)	26.04 (4.46)	27.36 (4.65)	1.06 (1.03; 1.09)	**1.06 (1.03; 1.09)**
Nodal OA, *n* (%)	225 (9.61)	188 (9.41)	37 (13.60)	1.52 (1.04; 2.21)	1.49 (0.99; 2.23)
N of comorbidities, mean (SD), *n* (%)	0.69 (0.79)	0.67 (0.77)	0.86 (0.90)	1.32 (1.14; 1.53)	**1.31 (1.12; 1.53)**
Any comorbidities, *n* (%)	1213 (51.82)	1039 (50.54)	174 (61.05)	1.53 (1.19; 1.98)	**1.48 (1.13; 1.95)**
Any comorbidities ≥ 2, *n* (%)	335 (14.30)	285 (13.86)	50 (17.54)	1.32 (0.95; 1.84)	1.26 (0.90; 1.78)
Any comorbidities ≥ 3, *n* (%)	62 (2.65)	46 (2.24)	16 (5.61)	2.60 (1.45; 4.66)	**2.54 (1.41; 4.60)**
Any comorbidities ≥ 4, *n* (%)	8 (0.34)	4 (0.19)	4 (1.40)	7.30 (1.82; 29.36)	**8.85 (1.95; 40.16)**

In bold - If a 95% confidence interval does not include the null value (i.e. there is a statistically meaningful or statistically significant difference between the groups).

**Abbreviations:** BMI - body mass index; KP – knee pain; OR – odds ratio; CI –confidence interval; HADS - Hospital Anxiety and Depression Scale; PCS - Pain Catastrophizing Scale; SD - standard deviation.

∗ Adjusted for gender and BMI only.

† Adjusted for age and BMI only.

‡ Adjusted for age and gender only.

**Table:** 

	Descriptive			OR (95% CI)	
	Total	Stable/improved	Worsened	Crude	Age, gender, BMI-adjusted
**N**	2396	1943	453		
**Central risk factors**					
Widespread pain, *n* (%)	794 (33.14)	594 (30.57)	200 (44.15)	1.80 (1.46; 2.21)	**1.68 (1.35; 2.10)**
HADS anxiety score ≥8, *n* (%)	900 (37.56)	678 (34.89)	222 (49.01)	1.79 (1.46; 2.20)	**1.68 (1.35; 2.08)**
HADS depression score ≥8, *n* (%)	551 (23.00)	394 (20.28)	157 (34.66)	2.09 (1.67; 2.61)	**1.85 (1.46; 2.34)**
PCS in the highest tertile (>=9), *n* (%)	998 (41.65)	726 (37.36)	272 (60.04)	2.52 (2.04; 3.11)	**2.27 (1.83; 2.83)**
mPDQ ≥ 19, *n* (%)	202 (8.43)	123 (6.33)	79 (17.44)	3.13 (2.31; 4.23)	**2.65 (1.92; 3.65)**
ICOAP intermittent in the highest tertile (>=7), *n* (%)	826 (34.47)	551 (28.36)	275 (60.71)	3.90 (3.15; 4.83)	**3.61 (2.89; 4.50)**
ICOAP constant in the highest tertile (>=7), *n* (%)	788 (32.89)	518 (26.66)	270 (59.60)	4.06 (3.28; 5.02)	**3.70 (2.96; 4.63)**
**Peripheral risk factors**					
Significant injury at baseline, *n* (%)	706 (29.47)	583 (30.80)	123 (28.21)	0.88 (0.70; 1.11)	0.89 (0.70; 1.13)
Significant injury in the past 12 months, *n* (%)	63 (2.63)	39 (2.01)	24 (5.31)	2.73 (1.62; 4.59)	**2.52 (1.48; 4.30)**
Early varus malalignment, *n* (%)	102 (4.26)	76 (4.23)	26 (6.31)	1.53 (0.96; 2.41)	1.60 (0.99; 2.58)
Early valgus malalignment, *n* (%)	59 (2.46)	44 (2.48)	15 (3.75)	1.54 (0.85; 2.79)	1.64 (0.89; 3.03)
Varus malalignment, *n* (%)	61 (2.55)	41 (2.21)	20 (4.67)	2.17 (1.26; 3.75)	**2.28 (1.30; 4.02)**
Valgus malalignment, *n* (%)	68 (2.84)	55 (2.96)	13 (3.04)	1.03 (0.56; 1.90)	0.71 (0.37; 1.37)
High risk occupation, *n* (%)	1093 (45.62)	867 (44.62)	226 (49.89)	1.24 (1.01; 1.52)	**1.29 (1.04; 1.60)**
2D4D ratio (type 3), *n* (%)	1188 (49.58)	979 (52.89)	209 (49.64)	0.88 (0.71; 1.09)	0.86 (0.69; 1.07)
**Other risk factors**					
Age (years), mean (SD)∗	62.41 (10.02)	62.26 (9.98)	63.03 (10.19)	1.01 (1.00; 1.02)	1.01 (1.00; 1.02)
Women, *n* (%)†	1397 (58.31)	1107 (56.97)	290 (64.02)	1.34 (1.09; 1.66)	1.34 (1.07; 1.66)
BMI (kg/m^2^), mean (SD)‡	28.29 (5.80)	27.93 (5.62)	29.83 (6.28)	1.05 (1.03; 1.07)	**1.05 (1.04; 1.07)**
Nodal OA, *n* (%)	364 (15.19)	269 (14.44)	95 (22.35)	1.71 (1.31; 2.22)	**1.62 (1.23; 2.14)**
N of comorbidities, mean (SD), *n* (%)	0.90 (0.90)	0.87 (0.89)	1.05 (0.91)	1.24 (1.11; 1.39)	1.13 (1.00; 1.27)
Any comorbidities, *n* (%)	1461 (60.98)	1152 (59.29)	309 (68.21)	1.47 (1.19; 1.83)	**1.29 (1.02; 1.64)**
Any comorbidities ≥ 2, *n* (%)	553 (23.08)	419 (21.56)	134 (29.58)	1.53 (1.22; 1.92)	1.26 (0.98; 1.61)
Any comorbidities ≥ 3, *n* (%)	127 (5.30)	99 (5.10)	28 (6.18)	1.23 (0.80; 1.89)	0.98 (0.62; 1.54)
Any comorbidities ≥ 4, *n* (%)	14 (0.58)	11 (0.57)	3 (0.66)	1.17 (0.33; 4.21)	0.70 (0.15; 3.18)

In bold - If a 95% confidence interval does not include the null value (i.e. there is a statistically meaningful or statistically significant difference between the groups).

**Abbreviations:** BMI - body mass index; KP – knee pain; OR – odds ratio; CI –confidence interval; HADS - Hospital Anxiety and Depression Scale; PCS - Pain Catastrophizing Scale; SD - standard deviation; mPDQ – modified Pain Detect Questionnaire; ICOAP - Measure of Intermittent and Constant Osteoarthritis Pain.

∗ Adjusted for gender and BMI only.

† Adjusted for age and BMI only.

‡ Adjusted for age and gender only.

**Table:** 

	Descriptive			OR (95% CI)	
	Total	Stable/improved	Worsened	Crude	Age, gender, BMI-adjusted
**N**	2396	1738	658		
**Central risk factors**					
Widespread pain, *n* (%)	794 (33.14)	561 (32.28)	233 (35.41)	1.15 (0.95; 1.39)	1.11 (0.92; 1.35)
HADS anxiety score ≥8, *n* (%)	900 (37.56)	642 (36.94)	258 (39.21)	1.10 (0.92; 1.32)	1.09 (0.90; 1.31)
HADS depression score ≥8, *n* (%)	551 (23.00)	398 (22.90)	153 (23.25)	1.02 (0.82; 1.26)	1.00 (0.80; 1.24)
PCS in the highest tertile (≥9), *n* (%)	998 (41.65)	722 (41.54)	276 (41.95)	1.02 (0.85; 1.22)	1.01 (0.84; 1.22)
mPDQ ≥ 19, *n* (%)	202 (8.43)	160 (9.21)	42 (6.38)	0.67 (0.47; 0.96)	0.63 (0.44; 0.91)
ICOAP intermittent in the highest tertile (≥7), *n* (%)	826 (34.47)	616 (35.44)	210 (31.91)	0.85 (0.71; 1.03)	0.81 (0.66; 0.99)
ICOAP constant in the highest tertile (≥7), *n* (%)	788 (32.89)	584 (33.60)	204 (31.00)	0.89 (0.73; 1.08)	0.82 (0.67; 1.00)
**Peripheral risk factors**					
Significant injury at baseline, *n* (%)	706 (29.47)	497 (29.30)	209 (32.45)	1.16 (0.95; 1.41)	1.18 (0.96; 1.44)
Significant injury in the past 12 months, *n* (%)	63 (2.63)	40 (2.31)	23 (3.51)	1.54 (0.91; 2.59)	1.43 (0.84; 2.44)
Early varus malalignment, *n* (%)	102 (4.26)	76 (4.73)	26 (4.32)	0.91 (0.58; 1.43)	0.89 (0.56; 1.42)
Early valgus malalignment, *n* (%)	59 (2.46)	42 (2.66)	17 (2.85)	1.08 (0.61; 1.91)	1.02 (0.57; 1.85)
Varus malalignment, *n* (%)	61 (2.55)	48 (2.89)	13 (2.08)	0.71 (0.38; 1.32)	0.66 (0.35; 1.27)
Valgus malalignment, *n* (%)	68 (2.84)	52 (3.13)	16 (2.56)	0.81 (0.46; 1.43)	0.80 (0.45; 1.43)
High risk occupation, *n* (%)	1093 (45.62)	764 (43.96)	329 (50.00)	1.27 (1.07; 1.53)	**1.30 (1.07; 1.56)**
2D4D ratio (type 3), *n* (%)	1188 (49.58)	854 (51.66)	334 (53.96)	1.10 (0.91; 1.32)	1.11 (0.92; 1.34)
**Other risk factors**					
Age (years), mean (SD)†	62.41 (10.02)	62.48 (9.96)	62.22 (10.20)	1.00 (0.99; 1.01)	1.00 (0.99; 1.01)
Women, *n* (%)‡	1397 (58.31)	997 (57.36)	400 (60.79)	1.15 (0.96; 1.38)	1.15 (0.96; 1.39)
BMI (kg/m^2^), mean (SD)§	28.29 (5.80)	28.21 (5.62)	28.50 (6.25)	1.01 (0.99; 1.02)	1.01 (0.99; 1.02)
Nodal OA, *n* (%)	364 (15.19)	248 (14.87)	116 (18.71)	1.32 (1.03; 1.68)	**1.34 (1.04; 1.72)**
N of comorbidities, mean (SD), *n* (%)	0.90 (0.90)	0.90 (0.91)	0.89 (0.86)	0.98 (0.89; 1.09)	0.95 (0.85; 1.06)
Any comorbidities, *n* (%)	1461 (60.98)	1053 (60.59)	408 (62.01)	1.06 (0.88; 1.28)	1.05 (0.86; 1.28)
Any comorbidities ≥ 2, *n* (%)	553 (23.08)	407 (23.42)	146 (22.19)	0.93 (0.75; 1.16)	0.88 (0.70; 1.10)
Any comorbidities ≥ 3, *n* (%)	127 (5.30)	99 (5.70)	28 (4.26)	0.74 (0.48; 1.13)	0.66 (0.42; 1.03)
Any comorbidities ≥ 4, *n* (%)	14 (0.58)	11 (0.63)	3 (0.46)	0.72 (0.20; 2.59)	0.46 (0.10; 2.07)

In bold - If a 95% confidence interval does not include the null value (i.e. there is a statistically meaningful or statistically significant difference between the groups).

**Abbreviations:** BMI - body mass index; KP – knee pain; OR – odds ratio; CI –confidence interval; HADS - Hospital Anxiety and Depression Scale; PCS - Pain Catastrophizing Scale; SD – standard deviation; mPDQ – modified Pain Detect Questionnaire; ICOAP - Measure of Intermittent and Constant Osteoarthritis Pain.

∗ A significant change from baseline was calculated using he least significant criterion (LSC) (Nguyen and Eisman, 2000, Wang *et al*., 2016).

† Adjusted for gender and BMI only.

‡ adjuSted for age and BMI only.

§ Adjusted for age and gender only.
